# Formation of various structures caused by particle size difference in colloidal heteroepitaxy

**DOI:** 10.1038/s41598-024-53850-2

**Published:** 2024-02-08

**Authors:** Masahide Sato

**Affiliations:** Emerging Media Initiative, Kanazawa University, Kanazawa, 920-1192 Japan

**Keywords:** Colloids, Self-assembly

## Abstract

By performing isothermal–isochoric Monte Carlo simulations with depletion force, the author investigated the dependence of the epitxial layer structure on the differences in the particle size between the substrate in colloidal heteroepitaxy. By changing the size of epitaxial particles and performing simulations comprehensively, various structures including the structures observed in a experiment, such as a honeycomb, one created by hexagonal heptamers, and one consisting of both pentagonal tiles and triangular tiles, were created. When the ratio of particle sizes between the epitxial layer and substrate takes a specific value, two types of hexagonal structures were created. One is the hexagonal layer parallel to the substrate layer and the other layer is rotated by 60^∘^ from the substrate layer. The former structure was created over a wide range of particle-size ratios, whereas the latter structure was created when the particle-size ratio was only around the specific ratio, and it seemed a metastable structure.

## Introduction

Colloidal crystals are promising functional materials of photonic crystals^1,2^, because the lattice distance can be tuned by controlling the size of the colloidal particle, and many lattice structures can be realized easily by modulating the interactions between the particles^3–6^. Many techniques have been used to create high quality crystals and various structures. For example, by using various sizes of colloidal particles and adding DNA strands to the particles, body-centered cubic (bcc), face-centered cubic (fcc), and other more complex structures have been created^7–9^. Adding patches to particles can create complex structures^10–17^. By using triblock patchy particles, which have two patches at their polar positions, structures such as cubic diamond, bcc and hexagonal tetrastack structures have been created via precursor clusters of tetrahedra and octahedra in three-dimensional systems^18–21^. In a simulation^22^, single colloidal gyroid structures, which provide a rich platform for chiroptics, were created for two distinct types of colloidal patchy sphere. The kagome lattice and other complex structures such as quasi-crystals have also been created in two-dimensional systems^23–27^.

Using templates or regular substrates for creating colloidal crystal, also known as colloidal epitaxy^28–32^, is also a useful method for creating high quality colloidal crystals. When monodisperse particles are solidified on flat walls by sedimentation, mixtures of fcc and hexagonal close-packed (hcp) structures are created because stacking faults form easily between the hexagonal layers. However, when the particles are solidified by sedimentation on a substrate with a square pattern, the growing interface becomes the (100) face of the fcc structure, and thick layers of the fcc structure can be created without inserting stacking faults. In addition to using patterned substrates and homoepitaxy technique, heteroepitaxy of colloidal particles^33–35^ has been also used to create many complex structures. Which types of structures are created and how epitaxial layers grow have been studied experimentally for both monodisperse^33,35^ and binary^34^ systems.

In this paper, by performing isothermal–isochoric Monte Carlo simulations, the dependence of the epitaxial layer structures on the radial size difference between the substrate particles and epitaxial particles was investigated. In simulations, the structure of the first epitaxial layer was mainly analyzed, because understanding the formation of the first epitaxial layer– the substrate of the growing crystal– is one of the most important points in creating high-quality materials by epitaxy. The formation of various structures in colloidal heteroepitaxy by controlling the particle sizes was studied comprehensively. Various structures including the structures observed in an experiment^35^ were found in our simulations. Because performing comprehensive experiments with various particle sizes takes long time and needs a lot of efforts, our simulation results are probably very helpful to investigate what kinds of structures can be created with different particle sizes and to make planes to create desired structures effectively.

## Results

In the Monte Carlo simulations, the depletion attraction was considered as the interaction between particles, and the Asakura-Osawa potential^36^ $U_\mathrm{AO}$ was used as the interaction potential. In the following simulations, $r_\mathrm{g}$ characterized the interaction length and $n_\mathrm{p}$ showed the strength of the interaction. All the simulations were performed under $U_\mathrm{AO}/k_\mathrm{B}T<1$, where $k_\mathrm{B}$ is the Boltzmann constant and *T* is temperature. In the simulations, particles with the radius $r_\mathrm{S}$ solidified on the closed-packed hexagonal structure of substrate particles with the radius $r_\mathrm{L}$, which were fixed to the bottom of the simulations box with the size $L_x \times L_y \times L_z$. Firstly, the relationship between the epitaxial layer structures and the particle sizes was examined. Then, the formation of two hexagonal layers created with a specific particle size ratio was studied.

### Relationship between the particle sizes and structure

**Figure 1:**
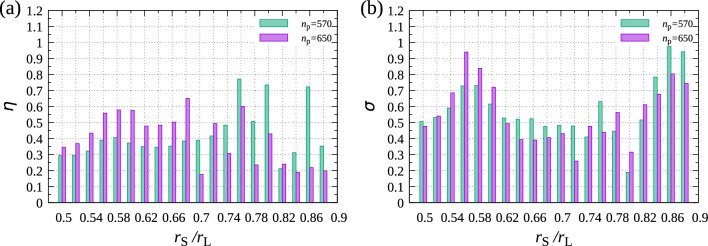
(**a**) Relationship between $r_\mathrm{S}/r_\mathrm{L}$ and the ratio of the surface area occupied by epitaxial particles to that by substrate particles, $\eta$. (**b**) Relationship between $r_\mathrm{S}/r_\mathrm{L}$ and the ratio of particles with $\phi _6>0.7$ to the particles attached to the surface, $\sigma$. In both figures, $n_\mathrm{p}=570$ and 650. For all the particle size ratios, the data are obtained from a single sample after $10^8$ trials for each particle. in which parameters are $r_\mathrm{L}=0.5$, $r_\mathrm{g}=0.025$, $N_\mathrm{L}=900$, and $N_\mathrm{S}=1800$.

**Dependence of coverage and** $\phi _6$ **on** $r_\mathrm{S}/r_\mathrm{L}$. Here, $r_\mathrm{S}/r_\mathrm{L}$ was changed over a broader range, and how the structure of epitaxial layer is related to $r_\mathrm{S}/r_\mathrm{L}$ was studied. Figure 1a indicates how much of the substrate was covered with epitaxial particles for various $r_\mathrm{S}/r_\mathrm{L}$. $\eta$ represents the ratio of the surface area occupied by epitaxial particles. The definition of $\eta$ is given by $N_\mathrm{S} \pi (r_\mathrm{S})^2 /L_x L_y$, where $N_\mathrm{S}$ is the number of epitaxial particles. $\eta$ was high for $r_\mathrm{S}/r_\mathrm{L}=0.86$, 0.80 and 0.76 when $n_\mathrm{p}=570$, which indicates that the epitaxial layer was well-fitted with the substrate layer. Compared with that with $n_\mathrm{p}=570$, $\eta$ decreased in the large $r_\mathrm{S}/r_\mathrm{L}$ region with $n_\mathrm{p}=650$, probably because three-dimensional islands were created in solution before attaching to the substrate at the large $n_\mathrm{p}$. However, $\eta$ increased with increasing $n_\mathrm{p}$ for small $r_\mathrm{S}/r_\mathrm{L}$. The attraction between the epitaxial particles and substrate was small, so that the epitaxial layer was hard to be created on the substrate when $r_\mathrm{S}$ was small. With increasing $n_\mathrm{p}$, the first epitaxial layer were created largely because the interaction energy became large even in the small $r_\mathrm{S}$ region. In particular, $\eta$ became large when $r_\mathrm{S}/r_\mathrm{L}=0.68$ and in the range of 0.56–0.6, which shows the good matching between the substrate and first epitaxial layer for these radii.

The epitaxial layer was affected by the symmetry of the substrate. Because the substrate was the hexagonal structure, structures with the six-fold rotational were expected to be created easily. Thus, to examine structures created on the substrate, the local six-fold rotational order $\phi _6$ was calculated. Figure 1b indicates how the ratio of the number of epitaxial particles with $\phi _6>0.7$ to that of all the particles interacting with the substrate, $\sigma$ is related to $r_\mathrm{S}/r_\mathrm{L}$. When $r_\mathrm{S}/r_\mathrm{L}=0.86$, 0.80 and 0.76, both $\sigma$ and $\eta$ increased with decreasing $n_\mathrm{p}$, whereas they increased with increasing $n_\mathrm{p}$ for $r_\mathrm{S}/r_\mathrm{L}=0.68$, 0.56, and 0.6. Because the particles forming epitaxial layer have large $\phi _6$ with these $r_\mathrm{S}/r_\mathrm{L}$, these changes in $\eta$ and $\sigma$ seem to indicate that the epitaxial layer with these $r_\mathrm{S}/r_\mathrm{L}$ formed a hexagonal lattice.

**Radial distribution function for** $r_\mathrm{S}/r_\mathrm{L}=0.74$–0.88. Detailed investigations are necessary to specify what structures were created, because other structures except for the hexagonal structure might have large $\phi _6$. Thus, to examine the long–range order, the radial distribution function *g*(*r*) was calculated. Figure S1 in Supplementary Information shows *g*(*r*) for $n_\mathrm{P}=570$ , in which $r_\mathrm{S}/r_\mathrm{L}$ changes from 0.74 to 0.88 each increment of 0.2. In each figure, the first peak is located at approximately $2r_\mathrm{S}$ in each figure. Because the interaction potential between epitaxial particles reaches a minimum when the particles attach to each other, the location of the first peak is reasonable. For $r_\mathrm{S}/r_\mathrm{L}=0.74$ ( Fig. S1a) and 0.78 (Fig. S1c), the peak locations are not clearer than others, which indicates that the long order were not created. For $r_\mathrm{S}/r_\mathrm{L}=0.84$ (Fig. S1f), 0.86 [(Fig. S1g)], and 0.88 (Fig. S1h), the second and third peaks are located at about $2\sqrt{3}r_\mathrm{S}$ and $4 r_\mathrm{S}$, respectively; consistent with formation of a hexagonal lattice. Hexagonal lattice was also created with $r_\mathrm{S}/r_\mathrm{L}=0.76$ (Fig. S1b). Manifestation of sharp peaks is evident in Fig. S1d, e, but the peak positions differ from those in Fig. S1b, f, g, and h, which suggests the formation of a structure that differs from a hexagonal structure.

**Figure 2:**
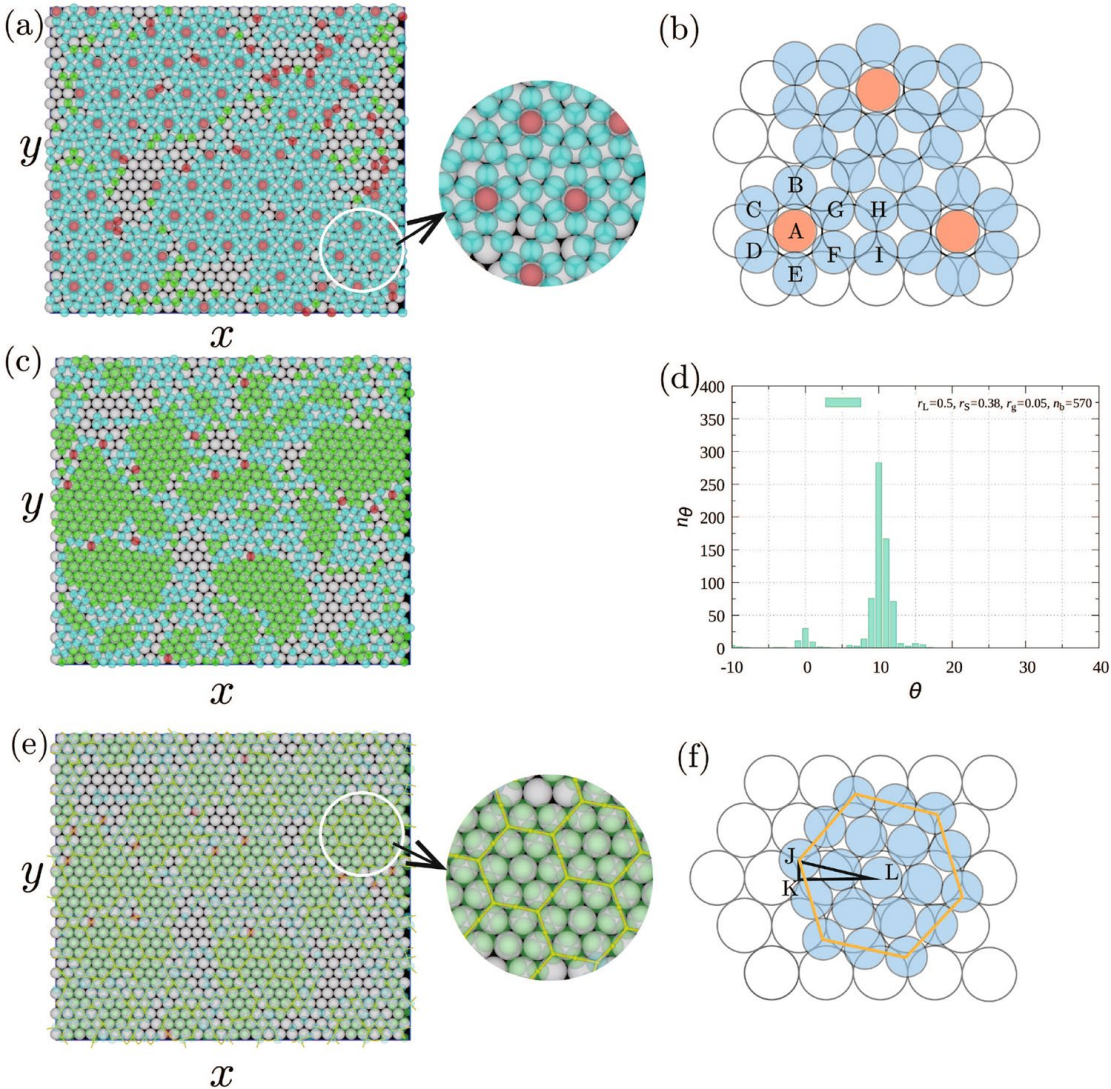
(**a**) Snapshot of the first epitaxial layers for $r_\mathrm{S}/r_\mathrm{L}=0.80$, (**b**) schematic of hexagonal heptamers, (**c**) snapshot of the first epitaxial layers for $r_\mathrm{S}/r_\mathrm{L}=0.76$, (**d**) the distribution of the tilting angle of the hexagonal structure created around particles with $\phi _6>0.7$ from the hexagonal structure created by the substrate, (**e**) snapshot in which the particles interacting with two substrate particles were connected by lines for (**e**) and (**f**) schematic of an ideal hexagonal tile. Circular figures adjacent to (**a**) and (**e**) are zoomed snapshot in circular areas. Parameters are $r_\mathrm{L}=0.5$, $r_\mathrm{g}=0.025$, $n_\mathrm{p}=570$, $N_\mathrm{L}=900$, $N_\mathrm{S}=1800$, $L_x=30.0$, $L_y=26.0$, and $L_z$ was changed with $r_\mathrm{S}$ for the particle density to be set at 0.1.

**Typical structures created for** $r_\mathrm{S}/r_\mathrm{L}=0.74$–0.88. To more directly confirm the manifested structures, typical snapshots for $r_\mathrm{S}/r_\mathrm{L}<1$ (Fig. 2) were checked. In the following figures, the substrate particles with radius $r_\mathrm{L}$ are white spheres. Particles that have $\phi _6>0.7$ are considered as particles in a hexagonal structure. Particles in a hexagonal structure rotated along the axis perpendicular to the substrate by approximately $15^\circ \sim 45^\circ$ are red, and those not rotated or rotated by less than 15^∘^ are green. The particles with $\phi _6<0.7$ are considered not to form the hexagonal structures and colored by light-blue. The particles which do not attach to the substrate are not drawn. When $r_\mathrm{S}/r_\mathrm{L}=0.8$ (Fig. 2a), instead of formation of hexagonal structures, heptamers with a hexagonal shape such as ABCDEFG in Fig. 2b, in which the center A is on the top of a substrate particle, were the unit of the structure. The heptamers were arranged regularly with two particles such as H and I placed between them. The center particles of the heptamers created a hexagonal lattice with the lattice constant $6 r_\mathrm{L}$. From Fig. 2b, the distance between the centers of two heptamers is approximately given by $2(2+ \sqrt{3} ) r_\mathrm{S}$ if small particles are assume to attach to each others on a flat plain. Thus, $r_\mathrm{S}/r_\mathrm{L}$ for creating this structure was approximately estimated to $3/(2+ \sqrt{3} )=0.803$, which roughly agreed with $r_\mathrm{S}/r_\mathrm{L}$ used in the simulations.

Considering $r_\mathrm{g}$, the structure shown in Fig. 2b can be created with epitaxial particles just barely attracting to each others when $r_\mathrm{S}/r_\mathrm{L}$ decreased to 0.76. However, from the form of Asakura-Osawa potential we used in the simulations, the interaction should become small with the distance. Thus, instead of the structure shown in Fig. 2b, a hexagonal structure was created again to gain much interaction energy between epitaxial particles (Fig. 2c). One primitive translation vector was tilted by $10^\circ$ from the substrate (Fig. 2d). When the particles interacting with two substrate particles were connected by lines (Fig. 2e), they formed hexagons, which were arranged periodically. Figure 2f shows a schematic of an ideal hexagonal tile. Because LJ$=4 r_\mathrm{S}$, KL$=3 r_\mathrm{L}$, and JK$=\sqrt{3}r_\mathrm{L}/3$, $r_\mathrm{S}$ and $\angle \textrm{JLK}$ were estimated to be $\sqrt{\textrm{KJ}^2 +\textrm{LK}^2}/4=0.764$ and $\tan ^{-1} (\textrm{JK}/\textrm{KL})= 10.89^\circ$, respectively. Thus, $r_\mathrm{S}$ used in the simulation and the tilting angle obtained from the simulation are consistent with the estimated values of an ideal hexagonal tile.

**Radial distribution function for** $r_\mathrm{S}/r_\mathrm{L}=0.50$–0.72. For $r_\mathrm{S}/r_\mathrm{L} \le 0.72$, *g*(*r*) and snapshots were examined with $n_\mathrm{p}=650$. Because the attraction between particles and substrates became small when $r_\mathrm{S}$ was small, larger $n_\mathrm{p}$ was used to create the epitaxial layer. Figure S2 in Supplementary Information shows *g*(*r*) from 0.50 to 0.72 each increment of 0.2. For $r_\mathrm{S}/r_\mathrm{L}=0.50$ (Fig. S2a), 0.52 (Fig. S2b), 0.62 (Fig. S2g), 0.64 (Fig. S2h) and 0.66 (Fig. S2i), sharp manifested peaks were not evident, which suggests that fine structures did not form with these radii. Because the peaks were located at the same positions when $r_\mathrm{S}/r_\mathrm{L}$ was in the range between 0.54 and 0.58 (Fig. S2c–e), the same structure, which is different from the hexagonal structure, was created with these $r_\mathrm{S}$. The peaks in Fig. S2d were sharpest among them, such that the structure was created most clearly with $r_\mathrm{S}/r_\mathrm{L}=0.56$. In other cases: Fig. S3f, j, k, l, the peaks in *g*(*r*) were sharp and $\sigma$ was large, which indicates that some structures different from the hexagonal structure were created as the first epitaxial layer.

**Figure 3:**
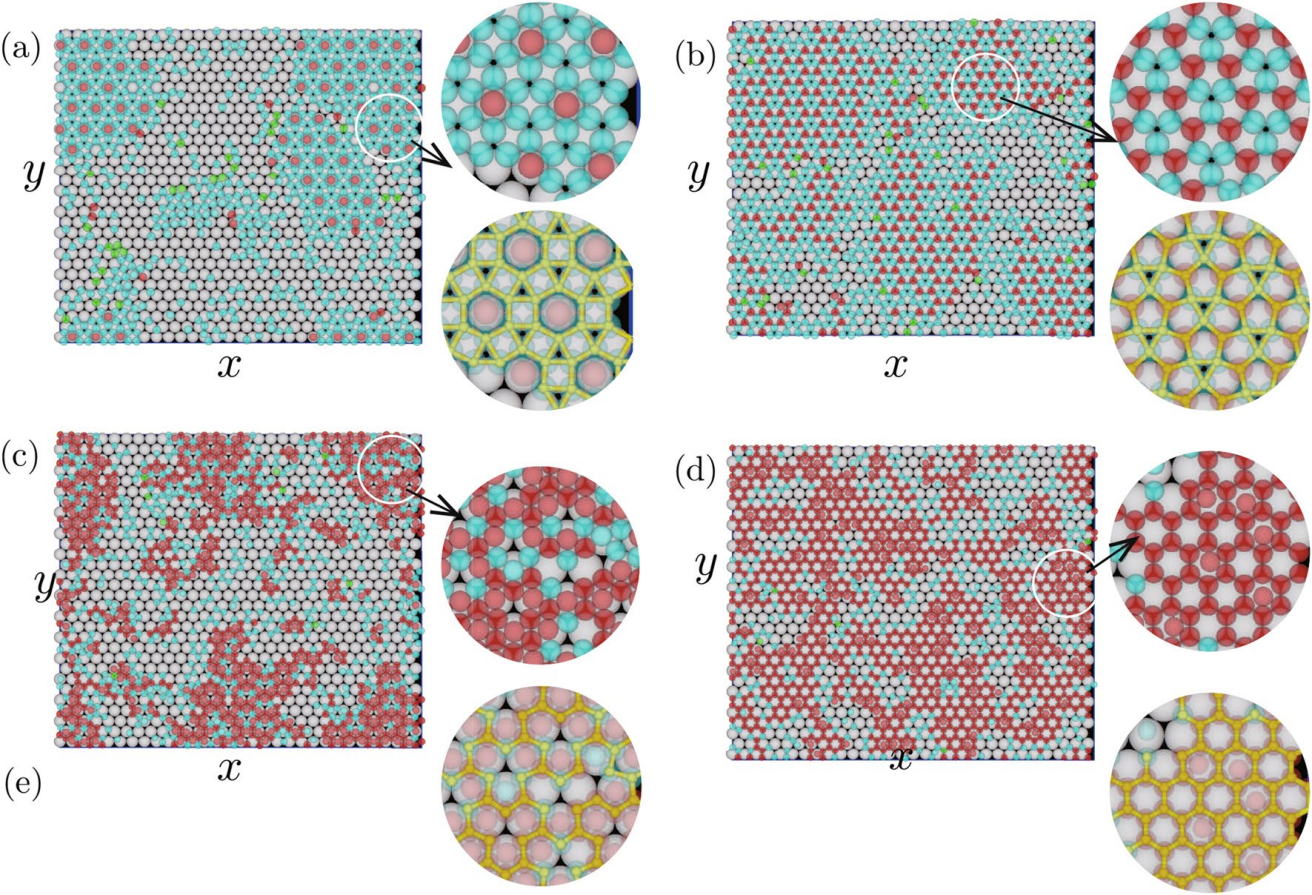
Snapshot of the first epitaxial layers for $r_\mathrm{S}/r_\mathrm{L}=$ (**a**) 0.72, (**b**) 0.68, (**c**) 0.60, and (**d**) 0.56. In each snapshot, two zoomed snapshots for circular area are drawn. In one of the zoomed snapshots, neighboring particles interacting with two or more substrate particles are connected with a yellow line. Parameters are $r_\mathrm{L}=0.5$, $r_\mathrm{g}=0.05$, $n_\mathrm{p}=650$, $N_\mathrm{L}=900$, $N_\mathrm{S}=1800$, $L_x=30.0$, $L_y=26.0$, and $L_z$ was changed with $r_\mathrm{S}$ for the particle density to be set at 0.1.

**Figure 4:**
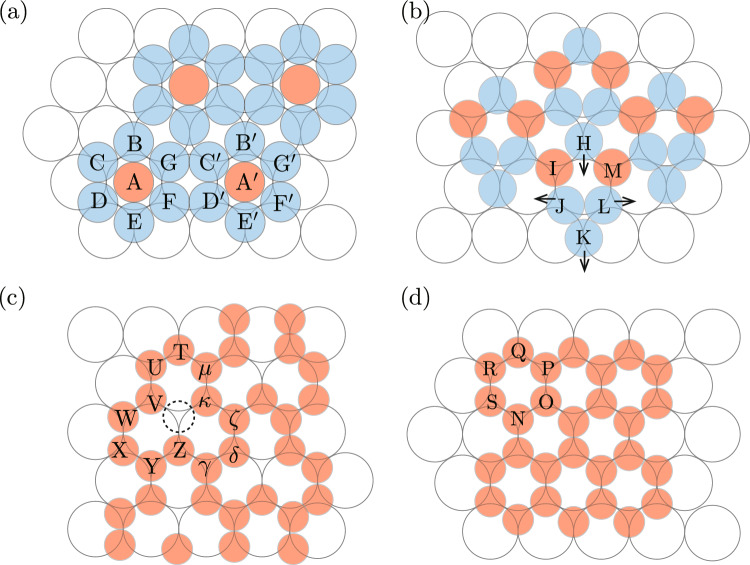
Schematics of the unit of structures for $r_\mathrm{S}/r_\mathrm{L}=$ (**a**) 0.72, (**b**) 0.68, (**c**) 0.60, and (**d**) 0.56.

**Typical structures created for** $r_\mathrm{S}/r_\mathrm{L}=0.50$–0.72. The structures expected from *g*(*r*) were confirmed from snapshots shown in Fig. 3. For $r_\mathrm{S}/r_\mathrm{L}=0.72$ (Fig. 3a), the hexagonal heptamers such as ABCDEFG and A^′^B^′^C^′^D^′^E^′^F^′^G^′^ shown in Fig. 4a were the units of the structure. The structure shown in Fig. 3a was similar to Fig. 2b although particles such as H and I, located between the hexagonal heptamers, were not evident. The centers of hexagonal heptamers such as $\textrm{A}$ and $\textrm{A}^\prime$ formed the hexagonal lattice with the lattice constant $4 r_\mathrm{L}$. Because the distance between A and A^′^ was approximately given by $2(1+\sqrt{3})r_\mathrm{S}$, $r_\mathrm{S}/r_\mathrm{L}$ was estimated to $2/(1+ \sqrt{3}) = 0.73$, which roughly agreed with the value used in simulations. As evident in Fig. 4a, the first epitaxial layer can also be regarded as the structure created by a triangular tile such as ABC and a square tile such as $\mathrm {FD^\prime C^\prime G}$. When $r_\mathrm{S}/r_\mathrm{L}=0.68$, the interaction between particles in the epitaxial layer can be weak if the structure shown in Fig. 3a is created. Thus, other structure, with which the interaction of particles in the epitaxial layer is strong and the matching with the substrate is good, was created (Fig. 3b). As evident in Fig. 4b, the first epitaxial layer was created by triangular tiles such as JKL, which consist of the particles interacting with two substrate particles, and pentagonal tiles such as HIJLM. They include particles interacting with two substrate particles (L, J and H) and those interacting with three substrate particles (M and I). When particle size is $r_\mathrm{S}/r_\mathrm{L}=0.60$, to increase the interaction with the substrate, the particles were located at the positions where particles interact with three substrate particles. Instead, the particles L and J cannot connect with each other. Because the particles H, J, L, and K, which connected with two substrate particles in Fig. 4b, moved toward the arrow direction, the structure consisting of a pentagonal tile and triangular tile changed the structure shown in Fig. 3c. $\textrm{TUVWXYZ}\gamma \delta \zeta \kappa \mu$ in Fig. 4c became the unit of the structure. When the particle size became smaller and $r_\mathrm{S}/r_\mathrm{L}=0.56$, the site shown by dotted circle in Fig. 4c were occupied by a particle and a honeycomb structure such as NOPQRS in Fig. 4d was created (Fig. 3d). The honeycomb structure was also observed for $r_\mathrm{S}/r_\mathrm{L}=0.58$, although the lattice was somewhat deformed. Because $r_\mathrm{s}$ should satisfy $2r_\mathrm{S}< 2 \sqrt{3} r_\mathrm{L}/3 < 2 (r_\mathrm{S} + r_\mathrm{g})$ for the honeycomb structure to be created, the range of $r_\mathrm{S}$ is estimated to $0.477< r_\mathrm{S}/r_\mathrm{L} < 0.577$ for the parameter we used, which is consistent with the simulation results.

The structures such as Fig. 3a, b were found in an experiment^35^. The diameters of the large and small particles in the experiment were $1300\textrm{nm}$ and $700\textrm{nm}$ for the honeycomb structure such as Fig. 3a, and $1100\textrm{nm}$ and $700\textrm{nm}$ for the structure shown in Fig. 3b. Thus, $r_\mathrm{S}/r_\mathrm{L}$ in the experiments is estimated to 0.54 and 0.634, respectively. These values in simulations are 1.04 and 1.07 times larger than the experimental values. The differences in $r_\mathrm{S}/\textrm{L}$ between the experiment and simulations was probably because of neglecting the electrostatic repulsion between particles in the simulations. However, because the difference was vary small, the simulations were consistent with the experiments.

**Figure 5:**
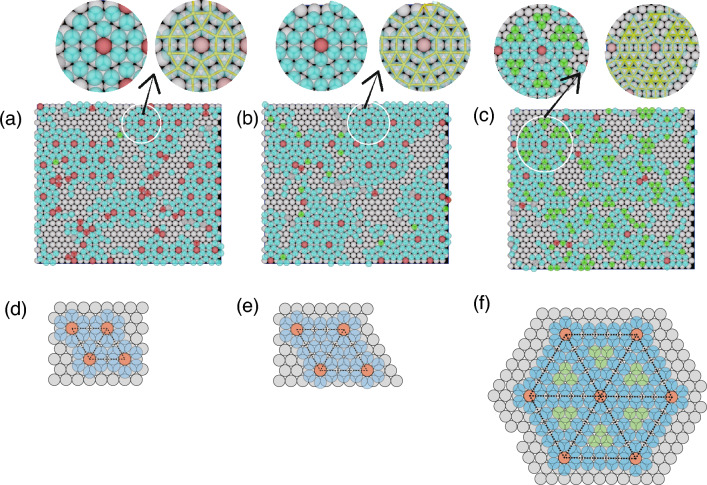
Snapshots of epitaxial layers for $r_\mathrm{S}/r_\mathrm{L}=$ (**a**) 1.08, (**b**) 1.06, and (**c**) 1.02. two zoomed snapshots for the circular area are drawn. In one of the zoomed snapshots, neighboring particles interacting with two or more substrate particles are connected with a yellow line. Parameters are $r_\mathrm{L}=0.5$, $r_\mathrm{g}=0.05$, $n_\mathrm{p}=500$, $N_\mathrm{L}=900$, $L_x=30.0$, $L_y=26.0$, and $L_z$ is set to $2r_\mathrm{L}+ 3r_\mathrm{S}$.

**Typical structures created for** $r_\mathrm{S}/r_\mathrm{L}>1$. When epitxial particles become larger than substrate particles, the particles prefer forming three-dimensional nuclei to attaching to the substrate and forming a two-dimensional structure because the interaction between large particles is strong in depletion force. Thus, to focus on what kinds of structures created in the first epitaxial layer, simulations were performed in a thin system in which the second layer is not created. Figure 5 shows snapshots created for epitxial particles slightly larger than the substrate particles, $r_\mathrm{S}/r_\mathrm{L}=1.08$, 1.06, and 1.04.

For $r_\mathrm{S}/r_\mathrm{L}=1.08$ (Fig. 5a), the unit of the structure was heptamer and the the centers of the heptamers made a triangular lattice as shown in Fig. 5d. The structure was similar to that for $r_\mathrm{S}/r_\mathrm{L}=0.72$, but the location of epitaxial particles on the substrate was different. The structure Fig. 5a was observed in an experiment^34^, but $r_\mathrm{S}/r_\mathrm{L}$ in the simulation is a little larger: the diameters of the substrate particles and the epitaxial particles are both $700\textrm{nm}$ in in the experiment, so that $r_\mathrm{S}/r_\mathrm{L}=1.0$. The difference in $r_\mathrm{S}/r_\mathrm{L}$ between the experiment and the simulations are roughly the same as those in Fig. 3a and b.

For $r_\mathrm{S}/r_\mathrm{L}=1.06$ (Fig. 5b), similarly to Fig. 5a, the unit of the structure is also a wheel-like structure, but the distance between the centers of the wheel-like structure is longer. When the difference in the particle size between the epitaxal layer and substrate becomes smaller, the unit of the structure became larger and more complex. For $r_\mathrm{S}/r_\mathrm{L}=1.04$ (Fig. 5c), the unity of the structure is not created perfectly in Fig. 5b but expected as Fig. 5f.

### Two epitaxial layers of hexagonal structures.

**Figure 6:**
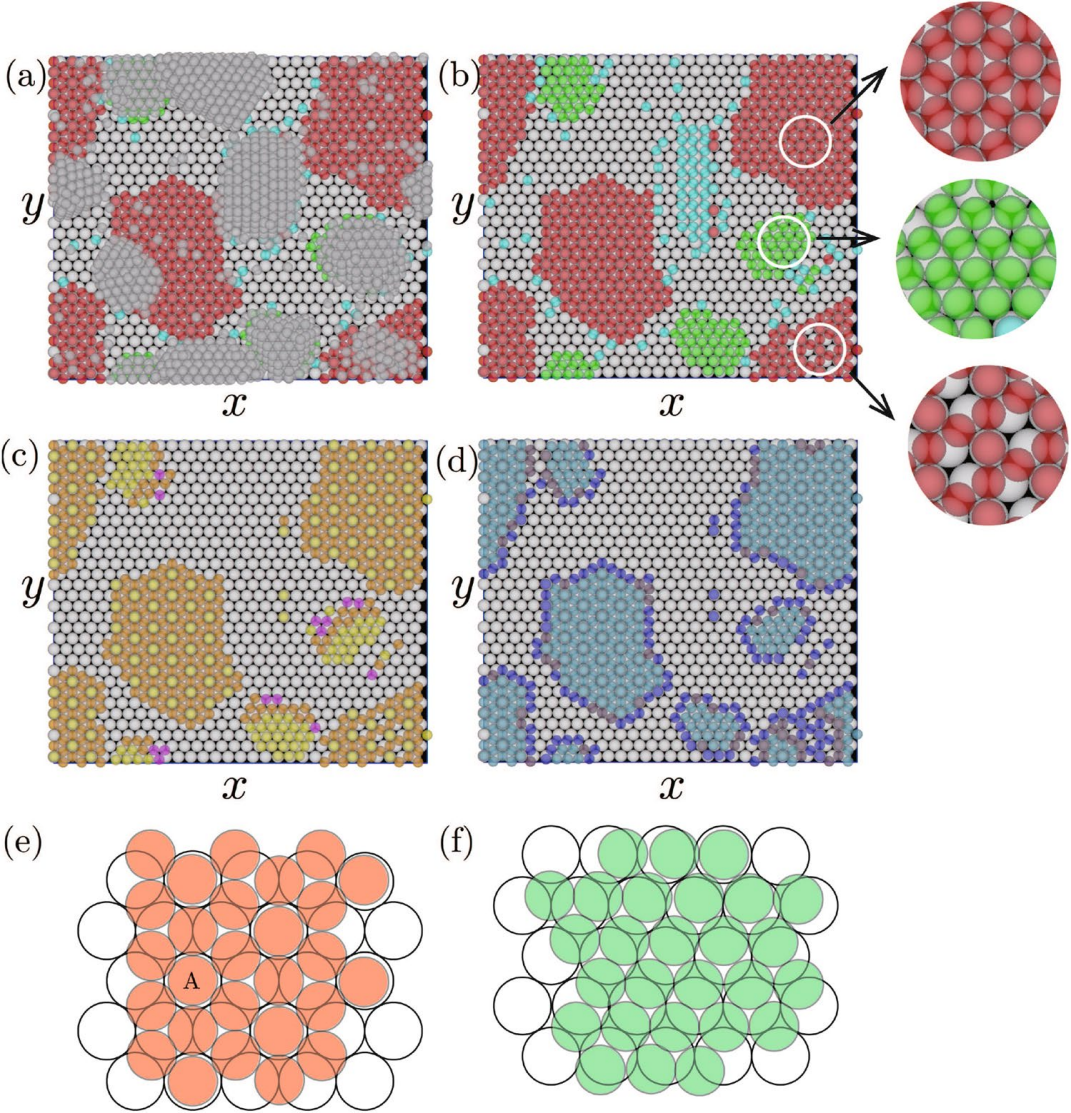
Snapshots of the epitaxial layer. The substrate particles are white. In snapshots (**a**) and (**b**), the particles in the hexagonal structures of $\alpha$ and $\beta$ structures are green and red, respectively. The criteria for determining the $\alpha$ and $\beta$ structures are the same as that for Fig. 2. In (**a**), the particles that do not interact with the substrate are gray. In (**c**), the number of interacting substrate particles is one for yellow spheres, two for orange spheres, and three for magenta spheres. In (**d**), the number of interacting particles in the first epitaxial layer is six for light-blue spheres, five for violet spheres, and four or three for blue spheres. Circular figures adjacent to (**b**) are zoomed snapshots for circular regions. In these snapshots, parameters are $r_\mathrm{L}=0.5$, $r_\mathrm{S}=0.43$, $r_\mathrm{g}=0.025$, $n_\mathrm{p}=560$, $N_\mathrm{L}=900$, $N_\mathrm{S}=2700$, $L_x=30.0$, $L_y=26.0$, and $L_z=12.1$. The particle density was set to 0.1. (**e**) and (**f**) Schematics of $\beta$ structure and $\alpha$ structure, respectively.

**Typical snapshots.** Two types of hexagonal structures were observed as the first epitaxial layer when $r_\mathrm{S}/r_\mathrm{L}=0.86$ (Fig. 6). Figure 6 shows snapshots of the epitaxial layers viewed from the *z*-direction. Hereafter, the hexagonal structure that is almost parallel to the substrate is termed $\alpha$ structure and that rotated along the axis perpendicular to the substrate by approximately $30^\circ$ is termed $\beta$ structure, respectively. Particles included in $\beta$ structure are red and those included in $\alpha$ structure are green. In addition to particles not included in the hexagonal structures, which are colored by light-blue, particles that do not interact with the substrate particles are drawn as gray spheres in Fig. 6a.

In Fig. 6c and d, the numbers of interacting substrate particles and interacting epitaxial particles in the first epitaxial layer are distinguished by color, respectively. Based on Fig. 6d, almost all the particles had six interacting epitaxial particles in the first epitaxial layers both in the $\alpha$ and $\beta$ structures, but the number of interacting substrate particles was different between the two structure in Fig. 6c. Particles interacting with one substrate particle and two particles were often evident in $\beta$ structure; the former particles were in hexagons created by the latter particles, whereas the arrangement of these two types of particles did not seem to exhibit obvious regularity in the $\alpha$ structure. In the $\beta$ structure, the *z*-coordinate of particle changed with a short distance. The height of the fist layer changes at the particle position, such as A in Fig. 6e. Compared with the $\beta$ structure, the height of the particle position seemed to change with a long distance in the $\alpha$ structure.

The difference between the $\alpha$ and $\beta$ structures in terms of the modulation in particle height caused the difference in solidification in the second and higher epitaxial layers on the two structures: the second and higher layers grew more easily on the $\alpha$ structure than on the $\beta$ structure (Fig. 6a). In the $\beta$ structure, because the large and sharp difference in the particle height was evident within a short period of distance, the growth of the second layer was prevented. In addition to a few three-dimensional islands, which seemed to form in the three-dimensional space by homogeneous nucleation, a three-dimensional island was created on the $\beta$ structure in the white circle in the right-bottom area in Fig 6a, but this three-dimensional island was formed on the first epitaxial layer with the vacancies (Fig 6b). The vacancies facilitated solidification of the particles on the first layer and the three-dimensional island was created.

In the $\alpha$ structure, the modulation of particle height was more gradual than in the $\beta$ structure, which did not prevent the formation of the second layer. Compared with the energy gain obtained by the formation of a large first layer of the $\alpha$ structure on the substrate, the energy gain by forming the second or higher epitaxial layers on the $\alpha$ structure was large, because particles in the first layer of the $\alpha$ structure interacted only with one or two substrate particles, although particles in the second layers were able to interact with three particles in the first layer. Thus, three-dimensional islands that are expected from Volmer–Weber (VM) growth mode were created on the $\alpha$ structure.

**Figure 7:**
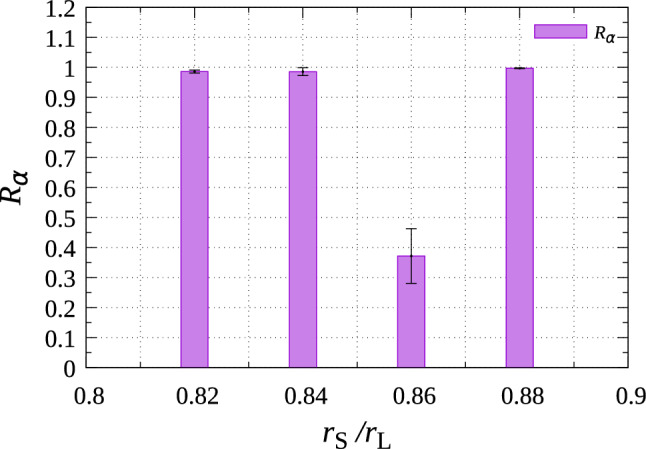
Ratio of the particle number in the $\alpha$ structure to that in hexagonal structures for $r_\mathrm{S}/r_\mathrm{L}=0.82 \sim 0.88$. The ratio was averaged over 30 individual runs. Parameters are $r_\mathrm{g}=0.025$, $n_p=560$, $N_\mathrm{L}=900$, $N_\mathrm{S}=2700$, $L_x=30.0$, $L_y=26.0$, and $L_z$ was changed to maintain the particle density at 0.1.

**Sensitivity of** $\beta$ **phase to the size of epitaxial particles.** The $\beta$ structure might be more sensitive to the difference between the epitxial layer and substrate regarding the particle sizes than $\alpha$ structure, because the $\beta$ structure was compatible with the substrate compared with the $\alpha$ phase. To confirm the difference in the sensitivity to the particle sizes, the dependence of the formation of the $\alpha$ and $\beta$ structures on the particle size was investigated. Figure 7 shows $R_\alpha$ for $r_\mathrm{S}/r_\mathrm{L}=0.82$, 0.84, 0.86, and 0.88, averaged over 30 individual runs. $R_\alpha$ was very close to unity for $r_\mathrm{S}/r_\mathrm{L}=0.82$, 0.84, and 0.88, whereas $R_\alpha <0.5$ for $r_\mathrm{S}/r_\mathrm{L}=0.86$. Namely, the $\beta$ structure was created just around $r_\mathrm{S}/r_\mathrm{L}=0.86$, because the matching between the epitaxial layer and substrate layer in the $\beta$ structure was much better than that in the $\alpha$ phase (Fig. 6).

Because the interactions between the particles acted in short-range, the range of $r_\mathrm{S}/r_\mathrm{L}$ in which the $\beta$ structure was created is estimated easily in accordance with the particle size. When the difference in the *z*-coordinates of the particles is neglected and the particles are assumed to be on a flat plane, the eptaxial particles create hexagonal lattices, in which the lattice constant is $2\sqrt{3}r_\mathrm{L}$. Because $r_\mathrm{S}$ and $r_\mathrm{L}$ must need to satisfy $2 r_\mathrm{S}< \sqrt{3} r_\mathrm{L} < 2 (r_\mathrm{S} + r_\mathrm{g})$ to create this hexagonal structure, the range of $r_\mathrm{S}$ forming the $\beta$ structure is estimated as $0.408<r_\mathrm{S}< 0.433$ for $r_\mathrm{L}=0.5$ and $r_\mathrm{g}=0.025$. The simulation results are approximately consistent with the estimation, although the lower limit of $r_\mathrm{S}/r_\mathrm{L}$ obtained from the simulation is somewhat restricted than this estimation.

**Stability of the** $\alpha$ **and** $\beta$ **structures.** The two hexagonal structures, the $\alpha$ and $\beta$ structures, are not equivalent from the free energy perspective. If longer simulations are performed, one of these structures should overcome the other. Because the simulations were preformed with $U_\mathrm{AO}/k_\mathrm{B}T <1$, as the first step, we examined which structure is more stable from the interaction energy perspective.

In Fig. 6a, higher layers were created on the $\alpha$ structure, while the layers were not created on the $\beta$ structure. Thus, for simplicity, we considered a monolayer of $\beta$ structure created by *N* particles and $\alpha$ structure created by *N*/2 particles, which is covered with the second layer with *N*/2 particles. In Fig. 6c, 57% of the particles in the $\alpha$ structure interacted with one substrate. The percentages of particles interacting with two particles and three particles are 31% and 12%, respectively. For the $\beta$ structure, 24% of particles such as A in Fig. 6e interacted with one substrate particle, and 76% of particles interacted with two substrate particles. Here, considering the particle ratios obtained from Fig. 6c, I estimated the energy gain by creating the $\alpha$ and $\beta$ structures. For simplicity, I assumed that for the $\alpha$ structure, the percentages of particles interacting with one particle, two particles, and three particles are given by 60%, 30% and 10%, respectively, and that for the $\beta$ structure, the percentages of particles interacting with one particle and two particles, are given by 25%, 75%, respectively. When the energy gain by the interaction between one epitaxial particle and one substrate particle is given by $\epsilon _0$, the interaction energy gains particle by attaching to substrate particles is given by $3N\epsilon _0/2$ for the $\alpha$ phase and $1.75N\epsilon _0$ for the $\beta$ phase. For the $\alpha$ phase, the energy gain by the interaction between the first layer and the second layer is necessary to be considered. As shown in Fig. S3 in Supplementary information, the particles in the second layer on $\alpha$ phase formed the hexagonal structure. Because each of them interacted with three particles in the first epitaxial layer, the energy gain per particle, which is given by the interaction between the first layer and second layers is given by $3N\epsilon _1/4$, where $\epsilon _1$ is interaction per particle between those layers. Thus, the total energy gain per particle for the $\alpha$ phase is $N(3\epsilon _0/2 + 3\epsilon _1)/2$. Because the particles seem to be very close enough to attach with each others in Fig. 6a, $\epsilon _0$ and $\epsilon _1$ are estimated as $4 \pi r_\mathrm{g}^2 r_\mathrm{S}r_\mathrm{L}/(r_\mathrm{S}+ r_\mathrm{L})$ and $2 \pi r_\mathrm{g}^2 r_\mathrm{S}$, respectively from Eq. (S5) in Supplementary Information. Because $N(3\epsilon _0/2 + 3\epsilon _1)/2 > 1.75N\epsilon _0$ when $r_\mathrm{S}/r_\mathrm{L} = 0.86$, $\alpha$ structure should overcome the $\beta$ phase and survive if longer simulations are performed.

**Figure 8:**
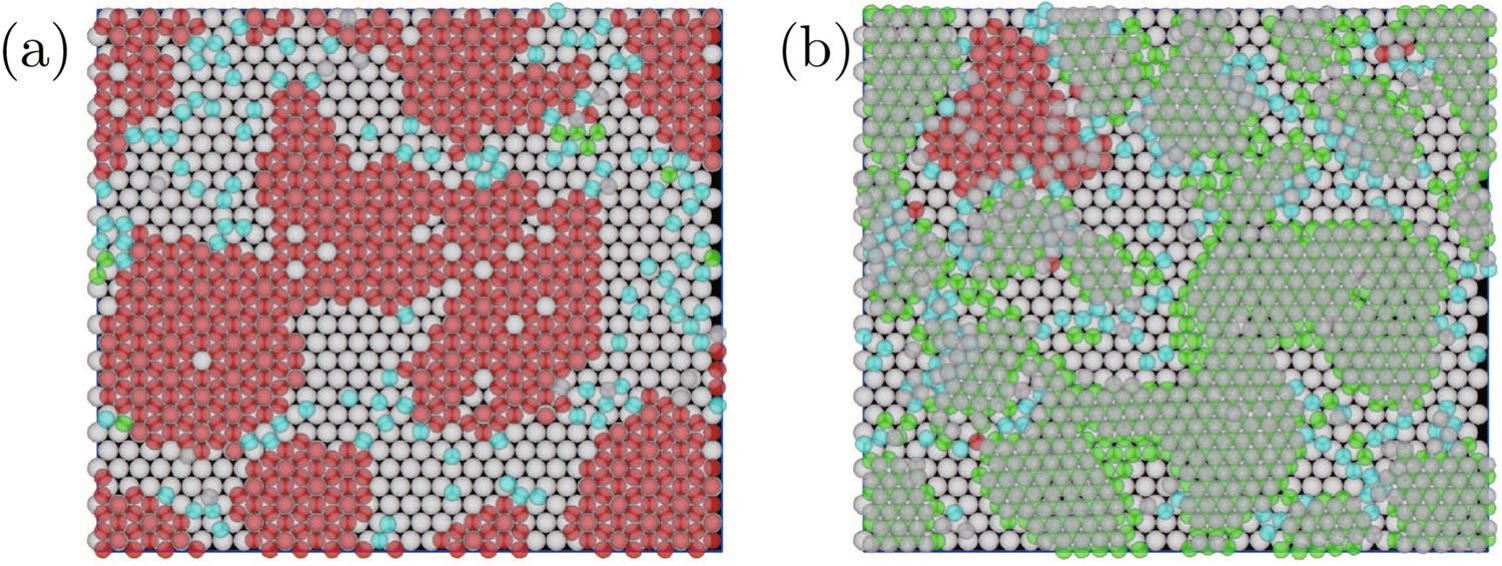
Snapshots of of epitaxial layers in thin systems. Parameters are $r_\mathrm{g}=0.025$, $n_p=560$, $N_\mathrm{L}=900$, $L_x=30.0$, $L_y=26.0$, $L_z$ and $N_\mathrm{S}$ are set to (**a**) $2r_\mathrm{L}+ 3 r_\mathrm{S}$ and 584, and (**b**) $2r_\mathrm{L}+ 5 r_\mathrm{S}$ and 1568, respectively.

Instead of performing long simulations, simulations with thin systems were performed and the stabilization of the $\alpha$ phase by the formation of the upper layers was confirmed. When the system is so thin that the formation of the second layer were not be able to created (Fig. 8a), the formation of the $\alpha$ phase was suppressed and the $\beta$ phase were created. However, when the system became thick enough for the second layer to be created, the $\beta$ phase became dominant (Fig. 8b). In Fig. S4 in Supplemental Information, the particle number in the $\alpha$ and $\beta$ structures, which is averaged over 20 individual runs, are shown. The result also shows that the formation of the second layer made the $\alpha$ phase more stable than the $\beta$ phase.

## Summary and discussions

By performing isothermal–isochoric Monte Carlo simulations, the dependence of the first heteroepitaxial layer of colloidal particles on the particle size difference between the epitaxial layer and substrate was investigated. In the simulations, the depletion attraction was considered as the interaction force acting between the particles. By changing the size of epitaxial particles, various complex structures including ones observed in an experiment^35^ were also created. In the simulations, the sizes of particles which created the structures observed in the experiment were slightly larger than those used in the experiment, but the differences were very small and negligible for considering the first epitaxial layer. Because performing comprehensive experiments with various particle sizes takes long time and needs a lot of efforts, the simulation results that showed what kinds of structures can be created with different particle sizes are probably very helpful to produce desired structures in experiments. In the simulations, nucleation on the upper wall were not observed although there were a few three-dimensional island, which were formed by homogeneous nucleation, and the height of the three-dimensional islands growing from the substrate was much smaller than $L_z$. Thus, effect of presence of the upper wall on the structures on the substrate is probably negligible.

When $r_\mathrm{S}/r_\mathrm{L}= 0.86$, two types of hexagonal structures, termed $\alpha$ structure, and $\beta$ structure, were created. In relation to the substrate, $\beta$ structure is rotated along the axis normal to the substrate by $30^\circ$ and $\alpha$ structure is not rotated. Three-dimensional islands that are expected from the VM growth mode were created on the $\alpha$ structure. Considering the poor matching between the epitaxial layer and substrate because of the fact that the number of interacting substrate particles per epitaxial particle was small, formation of islands as expected from the VM growth mode is reasonable. On the other hand, because of the good matching between the epitaxial layer and the substrate for the $\beta$ structure, this structure can spread substantially on the substrate. However, the second layer did not readily form on the $\beta$ structure, because the first epitaxial layer was bumpy, and the difference in the formation of the second layer made the $\alpha$ structure more stable than the $\beta$ structure.

In this study, only the depletion attraction was considered in the model, but in experiments^34,35,37^, other effects such as electric repulsion and van der Waals force probably affected the crystallization of colloidal crystals. It is not obvious whether these forces affect the structures of the upper layers on the various structure and the coexistence of the $\alpha$ structure and $\beta$ structure. In this paper, the structures of the epitaxial layer was examined. but the processes of the formation the epitaxial layer was not studied. In the simulations in this paper, the particle density was set to 0.1, but the particle density is the important parameter in crystallization^38^. If simulations are performed with various particles density by other simulations method such as Brownian dynamics simulation, not only the dependence of the structure of the first layer on the particle density but also the density dependence of the process of formation of the first layer can be examined. The author intends to study the growth processes of epitaxial layers and the effect of particle density.

## Methods

### Interaction potential

In simulations, the depletion attraction is considered, because this interaction is suggested to be one of the important interactions in an experiment^35^. The origin of the depletion interaction is the excluded volume effect caused by depletants in solution. In the simulations, particles and the depletion interaction were expressed as hard spheres and by the Asakura-Osawa potential^36^, respectively. The interaction potential between the *i*th and *j*th particles $U_\mathrm{OA}(r_{ij})$ is given by

1 $$\begin{aligned} U_\mathrm{OA}(r_{ij})&= {\left\{ \begin{array}{ll} \infty &{} (r_{ij}< 2 r_d) \\ -n_\mathrm{p}k_\mathrm{B}T V_\mathrm{OV}(r) &{} (2 r_d< r_{ij}< 2 R_d) \\ 0 &{} (2R_d < r_{ij} ) \end{array}\right. }, \end{aligned}$$

where $r_{ij}$ is the distance between the centers of the *i*th and *j*th particles. $n_\mathrm{p}$ represents the strength of the interaction, which increases with increasing the depletant density. Considering the dimension of Eq. (1), $n_\mathrm{p}$ is proportional to the depletant density. $k_\mathrm{B}$ is the Boltzmann constant, and *T* is temperature. When the radii of two particles are given by $r_i$ and $r_j$, $r_\mathrm{d}$ and $R_\mathrm{d}$ in Eq. (1) are expressed as

2 $$\begin{aligned} r_\mathrm{d}&=\frac{r_i+ r_j}{2}, \end{aligned}$$

3 $$\begin{aligned} R_\mathrm{d}&=\frac{r_i+ r_j }{2} + r_\mathrm{g}, \end{aligned}$$

where $r_\mathrm{g}$ characterizes the depletant size in solution. $V_\mathrm{OV}$ is the overlapping volume of two spherical regions, the radii of which are given by $r_i + r_\mathrm{g}$ and $r_j + r_\mathrm{g}$. The radii of the substrate particle and epitaxial particle are given by $r_\mathrm{L}$ and $r_\mathrm{S}$, respectively; the simulations were performed for $r_\mathrm{S}< r_\mathrm{L}$.

In Fig. S5a, the dependence of $U_\mathrm{OA}/k_\mathrm{B}T$ on the distance *r* between the particles are indicated for $r_\mathrm{L}=0.5$, $r_\mathrm{S}=0.43$, $r_\mathrm{g}=0.025$, and $n_\mathrm{p}=560$. The depletion force acts over a narrow range of *r*, monotonically increasing with decreasing *r*. Regarding the parameters used in Fig. S5a, the energy gain by the depletion attraction is a little larger than the thermal energy. The minimum of $U_\mathrm{OA}/k_\mathrm{B}T$ for two different size particles is slightly less than that for two larger size particles. Figure S5b indicates how the ratio of the potential minimum for two different size particles $U_\mathrm{min}^\mathrm{LS}$ to that for two large size particles $U_\mathrm{min}^\mathrm{LL}$ depends on the ratio of the two different particle sizes $r_\mathrm{S}/r_\mathrm{L}$. The ratio of these potential minima $U_\mathrm{min}^\mathrm{LS}/U_\mathrm{min}^\mathrm{LL}$ decreased with decreasing $r_\mathrm{S}/r_\mathrm{L}$; $U_\mathrm{min}^\mathrm{LS}/U_\mathrm{min}^\mathrm{LL}$ was approximately 0.93 when $r_\mathrm{S}/r_\mathrm{L}=0.86$.

### Simulation settings

Simulations were performed in a cuboidal system, the size of which is given by $L_x L_y L_z$; $L_x$, $L_y$, and $L_z$ are the sizes in the *x*-, *y*-, and *z*- directions, respectively. Two walls were located at $z=0$ and $z=L_z$. For the wall at $z=L_z$, the small particles acted as hard spheres with $r_\mathrm{S}$. The interaction between the wall and particles is the repulsion caused by the excluded volume effect. Periodic boundary conditions were used in the *x*- and *y*-directions. The system size was determined to make the particle density 0.1. The density used in the simulations was much larger than that used in an experiment^34^, but the density might be not so unrealistic, because of the gravitational sedimentation of particles during the experiment^34^, the particle density near the substrate was probably larger than the average particle density when the crystallization started.

Initially, $N_\mathrm{L}$ large particles with radius $r_\mathrm{L}=0.5$ were set on $z=0$ with the close-packed two-dimensional hexagonal structure, the lattice constant of which is $2r_\mathrm{L}$. The direction of one of primitive translation vectors was set parallel to the *x*-axis. $N_\mathrm{S}$ particles with radius $r_\mathrm{S}$ were located randomly in the system. The particles of radius $r_\mathrm{L}$ were fixed during the simulations, whereas the small particles were able to move in the systems. In the simulations, typical particle numbers with which simulations were performed were $N_\mathrm{L}=900$ and $N_\mathrm{S}=2700$ or 1800. The translation of particles was performed $2.5 \times 10^6$ times for each small particle without adding the attractive interactions to remove the effect of the initial configuration. Then, the attractive interactions were considered, and translation trials were performed $10^8$ times per small particle To prevent the acceptance ratio of trials being too small, the maximum migration length in a translation trial was tuned every 50 translation trials for all of the small particles, and the acceptance ratio was maintained at approximately 0.5 during the simulations.

### Local rotational order $\phi _{6}$

To evaluate the number of particles in the observed hexagonal structures quantitatively, the parameter for the six-fold orientational order, $\phi _{6}$, was calculated. The definition of $\phi _{6}$ for the *i*th particle $\phi _{6}(i)$ is given by

4 $$\begin{aligned} \phi _{6}(i)&= \frac{1}{N_\mathrm{B}(i)} \left| \sum _j \exp \left( 6 \textrm{i} \theta _{ij} \right) \right| , \end{aligned}$$

where $N_\mathrm{B}(i)$ is the number of neighboring particles for the *i*th particle, and $\theta _{ij}$ is the angle created by the *x*-axis and the line connecting the *i*th and *j*th particles. When the distance between the centers of the ith and jth particles was smaller than $2R_g$, these two particles as neighbors were regarded as the neighbors. The summation was performed for all neighboring particles when the *i*th particle had more than two neighbors. The threshold of $\phi _6$ for judging the formation of hexagonal structure involves a degree of arbitrariness. When $\phi _{6}(i) \ge 0.7$, the *i*th particle was identified as a particle included in a hexagonal structure.

### Radial distribution function

The definition of *g*(*r*) is given by

5 $$\begin{aligned} g(r)&= \frac{ \overline{n}(r)}{2 \pi r \Delta r \rho _{xy}}, \end{aligned}$$

In Eq. (5), where $\rho$ is the density of particles interacting with the substrate, and $\overline{n}(r)$ is the number of particles between distances *r* and $r+\Delta r$ attaching to the substrate, averaged over all the particles in one sample.

### Supplementary Information

Supplementary Information.

## Data Availability

The data that support the plots within this paper and other findings of this study are available from the corresponding authors upon reasonable request.

## Code availability

The codes that support the plots within this paper and other findings of this study are available from the corresponding authors upon reasonable request.
