# Supplementary material for: Formation of various structures caused by particle size difference in colloidal heteroepitaxy
Source: Sci Rep. 2024 Feb 8;14:3245. doi: 10.1038/s41598-024-53850-2 (PMC10853232; doi:10.1038/s41598-024-53850-2)
Supplement: Supplementary file 1 — Supplementary Information. [file 41598_2024_53850_MOESM1_ESM.pdf]

Supplymentary Information: Formation of Various Structures Caused by Particle Size Difference in Colloidal Heteroepitaxy

Masahide, Sato[1,*]

[1]Emerging Media Initiative, Kanazawa University, Kanazawa 920-1192, Japan
[*]msato002@staff.kanazawa-u.ac.jp

ABSTRACT

Figure S1 : $g(r)$ for $r_S/r_L = 0.74 \sim 0.88$.
Figure S2: $g(r)$ for $r_S/r_L = 0.50 \sim 0.72$.
Figure S3: Snapshot of the second layer on β structure for Fig. 6(a) .
Figure S4:Dependence of the system thickness on the formation of α and β structures
Figure S5: Form of $U_{OA}/k_B T$
Equation (S5) in Asakura-Osawa potential: the minimum of the Asakura-Osawa potential.

1 Asakura-Osawa potential

When polymers are added in solution, depletion force is caused by the polymers when particle distance is small enough for the polymers cannot penetrate the space between the particles. Asakura-Osawa potential often used as the potential for depletion force, U_{AO} is given by

$$U_{AO}(r) = \begin{cases} \infty & (r_{ij} < 2r_d) \\ -n_p k_B T V_{OV}(r) & (r_i + r_j < r < r_i + r_j + 2r_g) \\ 0 & (r_i + r_j + 2r_g < r) \end{cases}, \tag{S1}$$

where n_p is the polymer particle density, k_B is the Boltzmann constant, T is temperature, r_g is the radius of gyration of the polymer in water, and r_i and r_j are the radii of the ith and jth particles, respectively, and r is the distance between the centers of the two particles. $V_{OV}(r)$ indicates the overlap volume of the two spheres, the radii of which are given by $r_i + r_g$ and $r_j + r_g$, and is expressed as

$$V_{OV}(r) = \frac{\pi(r_i + r_g)^3}{3}(2 + \cos\theta_i)(1 - \cos\theta_i)^2 + \frac{\pi(r_j + r_g)^3}{3}(2 + \cos\theta_j)(1 - \cos\theta_j)^2, \tag{S2}$$

where $\cos\theta_i$ and $\cos\theta_j$ are given by

$$\cos\theta_i = \frac{(r_i + r_g)^2 + r^2 - (r_j + r_g)^2}{2(r_i + r_g)r}, \tag{S3}$$

$$\cos\theta_j = \frac{(r_j + r_g)^2 + r^2 - (r_i + r_g)^2}{2(r_j + r_g)r}. \tag{S4}$$

When $r = r_i + r_j$, the overlap of the spheres gets maximum and the potential $U_{AO}(r)$ has its minimum. When r_g is much smaller than r_i and r_j, the potential minimum $U^{ij}_{\min}$ is given by

$$U^{ij}_{\min} = -4\pi n_p k_B T r_g^2 \frac{r_i r_j}{r_i + r_j}. \tag{S5}$$

Radial distribution function

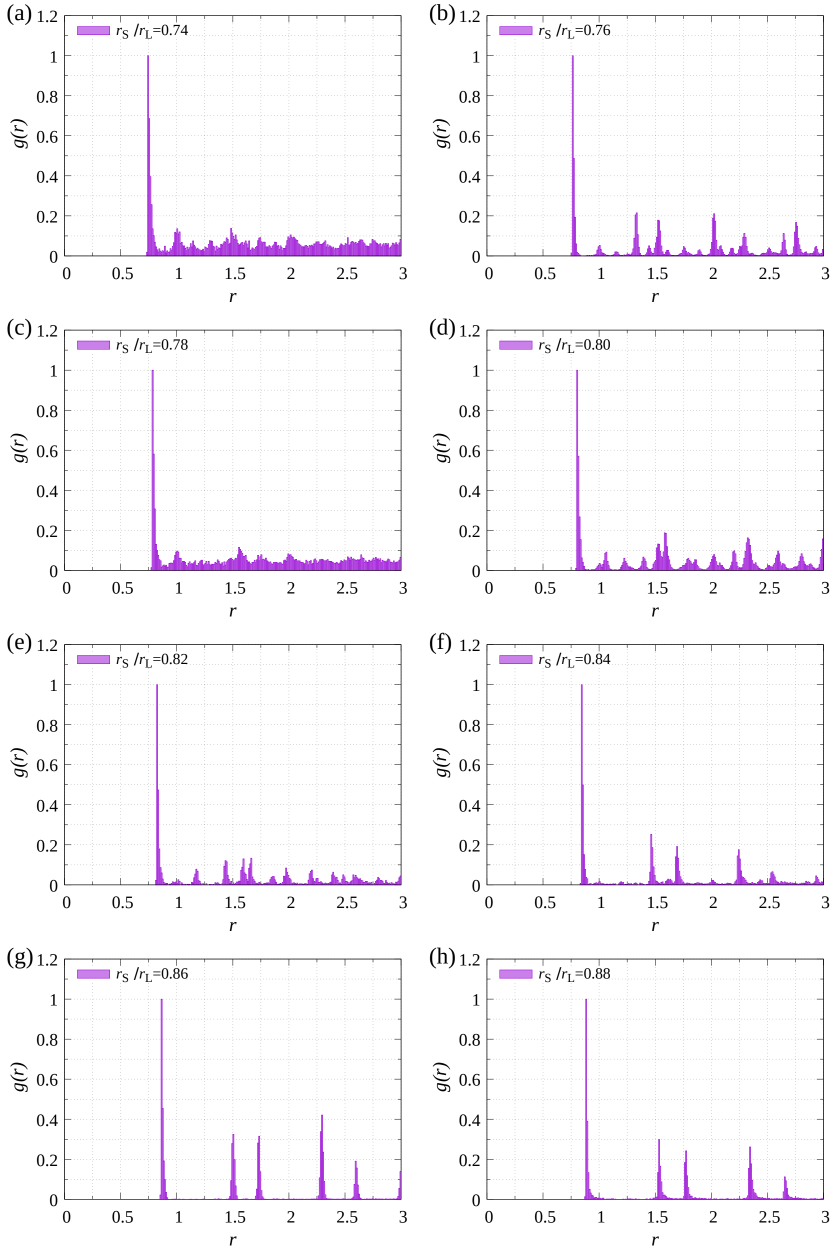

Figure S1. Radial distribution function $g(r)$ normalized by the highest peak for $n_P = 570$, in which r_S/r_L changed from 0.74 to 0.88 each increment of 0.2.

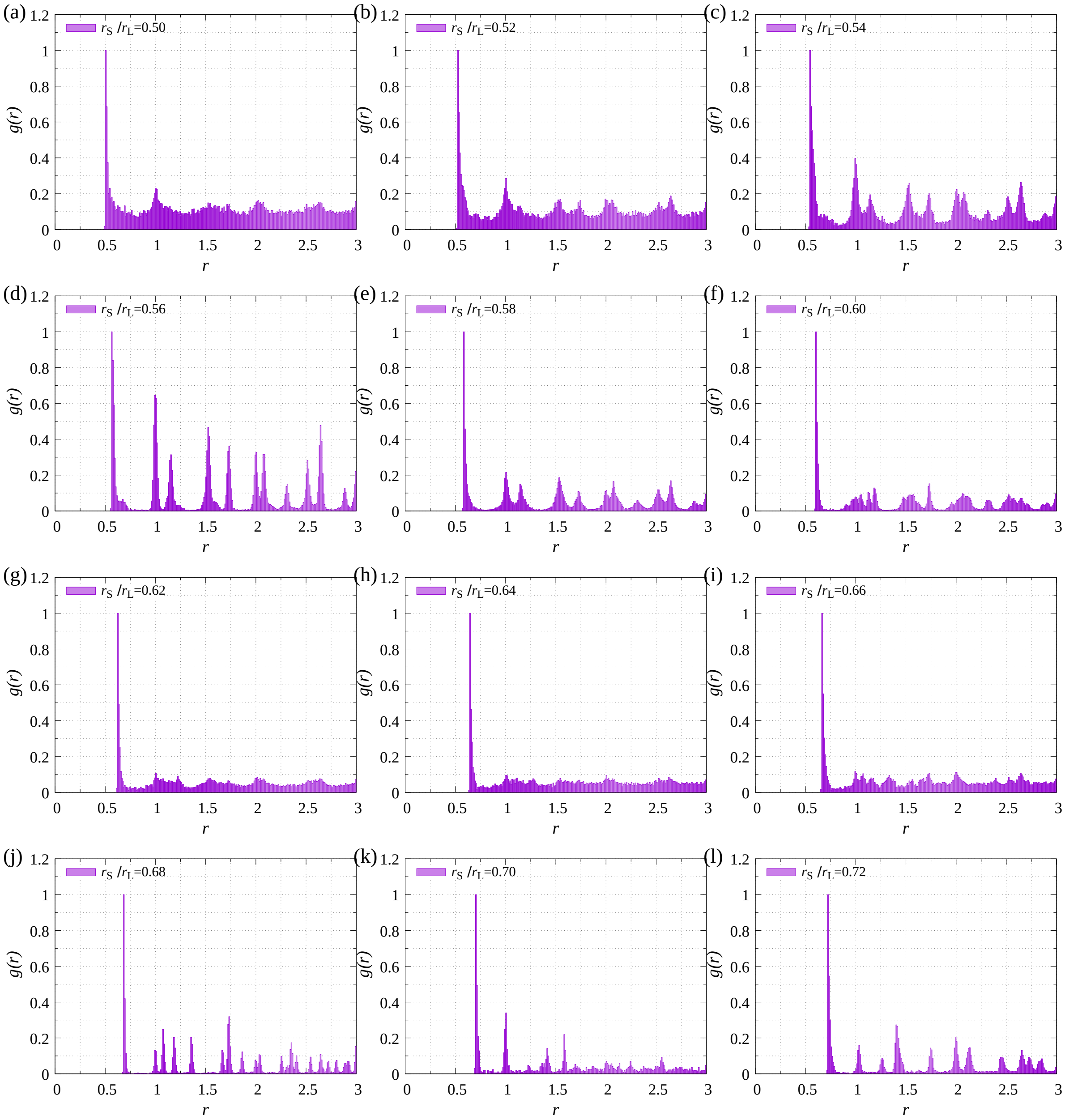

Figure S2. Radial distribution function $g(r)$, normalized by the highest peak. $r_\mathrm{S}/r_\mathrm{L}$ was changed from 0.50 to 0.72 each increment of 0.2

Snapshot of the second layer on α structure

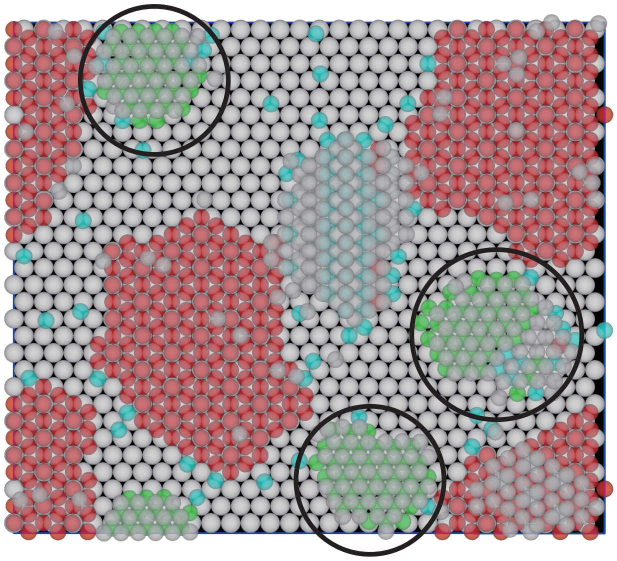

Figure S3. Snapshot of the second layer on α structure for Fig. 6(a). The the second layer on α structure is shown the area surrounded by black circles.

Dependence of the system thickness on the formation of α and β structures

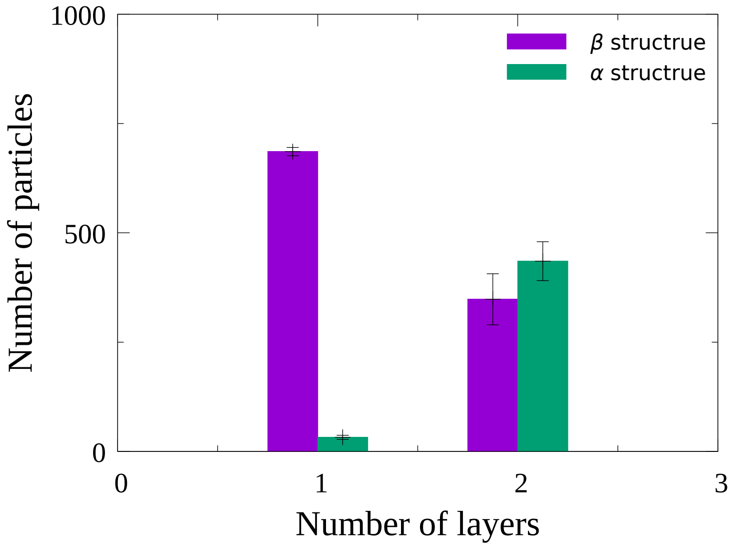

Figure S4. Dependence of the system thickness on the formation of α and β structures, which is averaged over 20 individual runs.

From of $U_{\mathrm{OA}}/k_{\mathrm{B}}T$

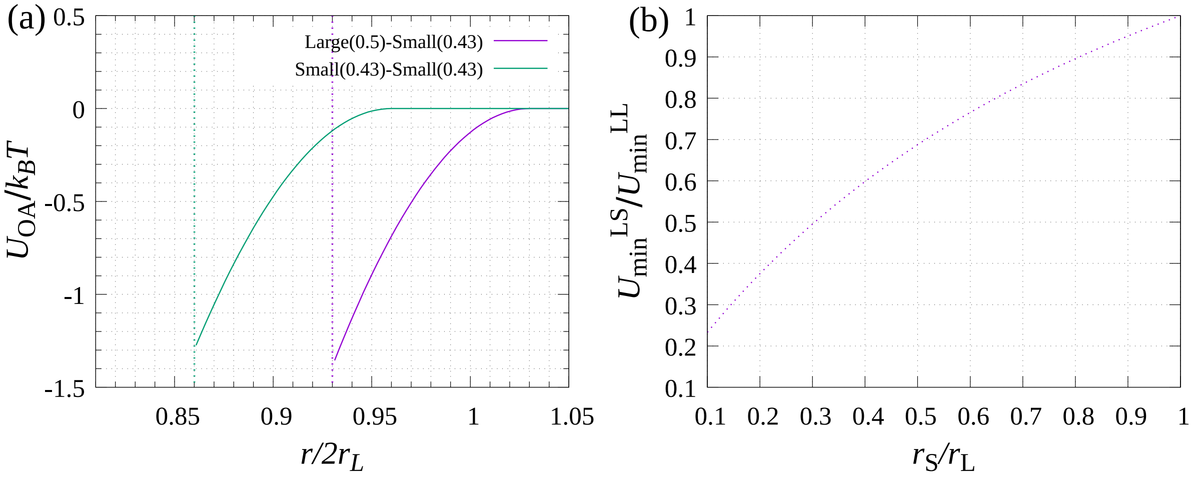

Figure S5. (a) Dependence of $U_{\mathrm{OA}}/k_{\mathrm{B}}T$ on r for two different-size particles and two small-size particles; where $r_{\mathrm{L}} = 0.5$, $r_{\mathrm{S}} = 0.43$, $r_{\mathrm{g}} = 0.025$, and $n_{\mathrm{p}} = 560$. (b) Dependence of the ratio of the depth of the potential minimum to that for two large-size particles $U_{\mathrm{min}}^{\mathrm{LS}}$ on the ratio of their sizes $r_{\mathrm{S}}/r_{\mathrm{L}}$.
